# Machine Learning Algorithms for understanding the determinants of under-five Mortality

**DOI:** 10.1186/s13040-022-00308-8

**Published:** 2022-09-24

**Authors:** Rakesh Kumar Saroj, Pawan Kumar Yadav, Rajneesh Singh, Obvious.N. Chilyabanyama

**Affiliations:** 1 Department of Community Medicine, Sikkim Manipal Institute of Medical Sciences-Sikkim Manipal University, Gangtok, Sikkim 737102 India; 2 Department of Biostatistics and Epidemiology, International Institute for Population Sciences, Mumbai, 400088 India; 3 Department of Mathematics and Statistics, Banasthali Vidyapith, Vanasthali Rd, Aliyabad, Tonk, Rajasthan 304022 India; 4 Centre for Infectious Disease Research in Zambia, Lusaka, Zambia; 5 African Centre of Excellency in Data Science (ACEDS), University of Rwanda, KK 737 Street, Gikondo, Kigali, Rwanda

**Keywords:** Under-five mortality, Machine learning, Random Forest, Neural Network, Accuracy

## Abstract

**Background:**

Under-five mortality is a matter of serious concern for child health as well as the social development of any country. The paper aimed to find the accuracy of machine learning models in predicting under-five mortality and identify the most significant factors associated with under-five mortality.

**Method:**

The data was taken from the National Family Health Survey (NFHS-IV) of Uttar Pradesh. First, we used multivariate logistic regression due to its capability for predicting the important factors, then we used machine learning techniques such as decision tree, random forest, Naïve Bayes, K- nearest neighbor (KNN), logistic regression, support vector machine (SVM), neural network, and ridge classifier. Each model’s accuracy was checked by a confusion matrix, accuracy, precision, recall, F1 score, Cohen’s Kappa, and area under the receiver operating characteristics curve (AUROC). Information gain rank was used to find the important factors for under-five mortality. Data analysis was performed using, STATA-16.0, Python 3.3, and IBM SPSS Statistics for Windows, Version 27.0 software.

**Result:**

By applying the machine learning models, results showed that the neural network model was the best predictive model for under-five mortality when compared with other predictive models, with model accuracy of (95.29% to 95.96%), recall (71.51% to 81.03%), precision (36.64% to 51.83%), F1 score (50.46% to 62.68%), Cohen’s Kappa value (0.48 to 0.60), AUROC range (93.51% to 96.22%) and precision-recall curve range (99.52% to 99.73%). The neural network was the most efficient model, but logistic regression also shows well for predicting under-five mortality with accuracy (94% to 95%)., AUROC range (93.4% to 94.8%), and precision-recall curve (99.5% to 99.6%). The number of living children, survival time, wealth index, child size at birth, birth in the last five years, the total number of children ever born, mother’s education level, and birth order were identified as important factors influencing under-five mortality.

**Conclusion:**

The neural network model was a better predictive model compared to other machine learning models in predicting under-five mortality, but logistic regression analysis also shows good results. These models may be helpful for the analysis of high-dimensional data for health research.

## Introduction

**Fig. 1:**
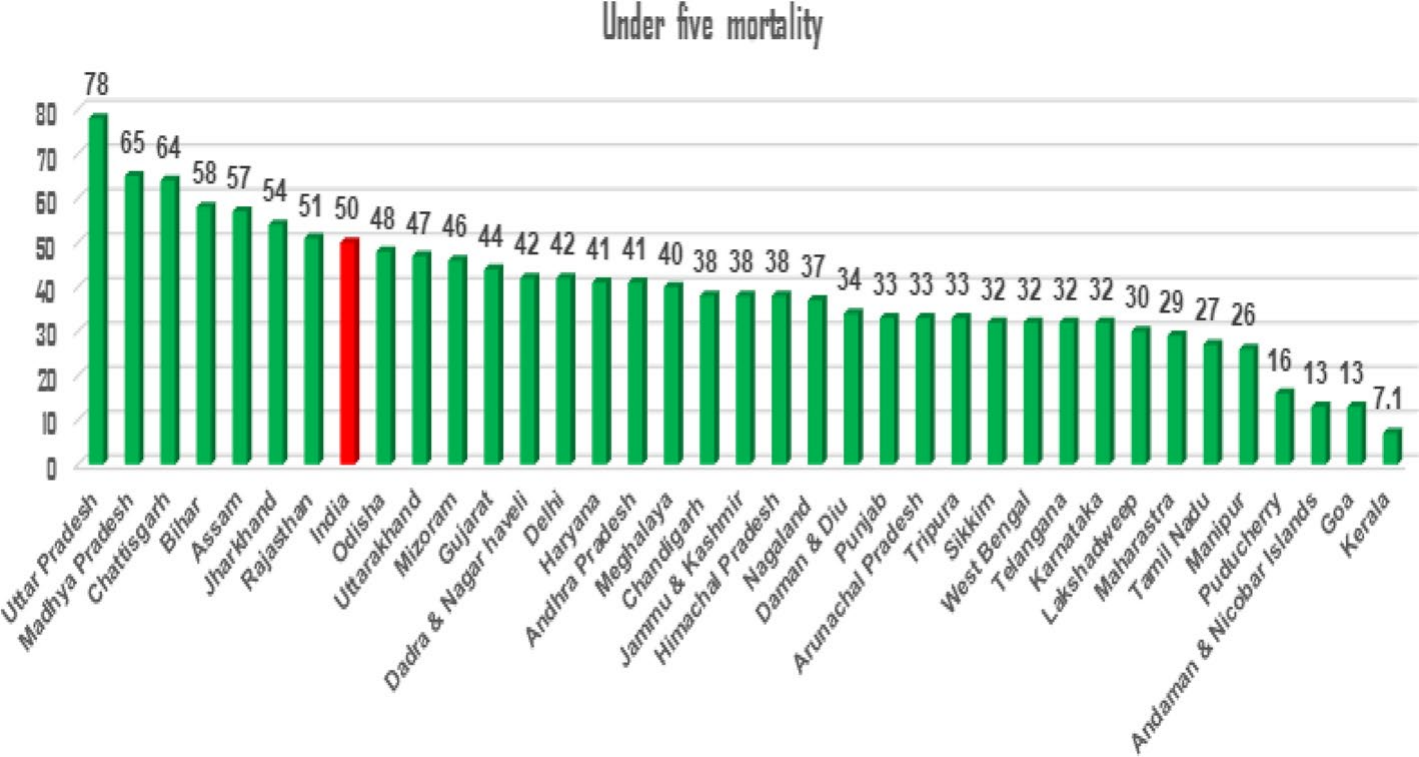
Under-five mortality of Uttar Pradesh comparison graph of state-wise from (NFHS-4)

Under-five mortality is the most widely used indicator to measure the health status of children. It is also an index of the general development of any country. Under-five mortality is the probability of children dying before their fifth birthday. Worldwide, under-five mortality rates are higher in the South-Asian and Sub-Saharan African countries. In India, the under-five mortality rate has reduced from 83 deaths per 1000 live births in 2000 to 42 deaths in 2017 [1]. State-wise reports have found that under-five mortality is highest in Uttar Pradesh, followed by Madhya Pradesh and Chhattisgarh [2], as shown in Fig. 1. Although there has been a significant reduction in under-five deaths in these states, it remains a major issue for child health in developing countries like India. Understanding the important factors in explaining childhood mortality is integral to reducing the death rate, but it is not enough.

Nowadays, Machine learning (ML) techniques are highly used in public health research. Various machine learning models have been used to predict and classify various health and biomedical data. These ML models can automatically identify interactions and find the non-linear relationship between the target variable and independent variables. Machine learning approaches can be utilized to discover the exposures related to health outcomes of interest and the potential interactions between those exposures [3]. Various machine learning prediction and classification models like regression, logistic regression, principal component analysis (PCA), decision trees, and maximum likelihood methods have been used to find the accurate estimation of health data. These approaches could help to obtain early prediction and insight into the important factors for under-five mortality. A study by Ethiopian provides evidence of J48 machine learning and artificial neural network (ANN) techniques to find the causes of child mortality [4]. Another study showed that the machine learning model effectively predicted the under-nutrition status of under-five children in the Ethiopian administrative zones [5]. The studies assessed the machine learning technique’s performance to predict the risk of neonatal mortality and morbidity [6, 7]. A study used iterative dichotomiser3(ID3), random forest, and decision tree models to predict the nutritional status of under-five children [8]. Another Indian study predicted the nutrient effects on human health using machine learning techniques [9]. So far in our literature search, no published study which used the machine learning model technique to predict under-five mortality was available. Also, past studies have found a lack of a generic prediction framework for accurately estimating child mortality rates using machine learning techniques.

There is a need for accurate prediction and classification models to provide highly accurate results and allow health researchers to experiment with various sets of aspects. This study offers an opportunity to assess the accuracy or efficacy of the machine learning models and find the important factors with the help of the information gain method in studying under-five mortality.

## Methodology

**Fig. 2:**
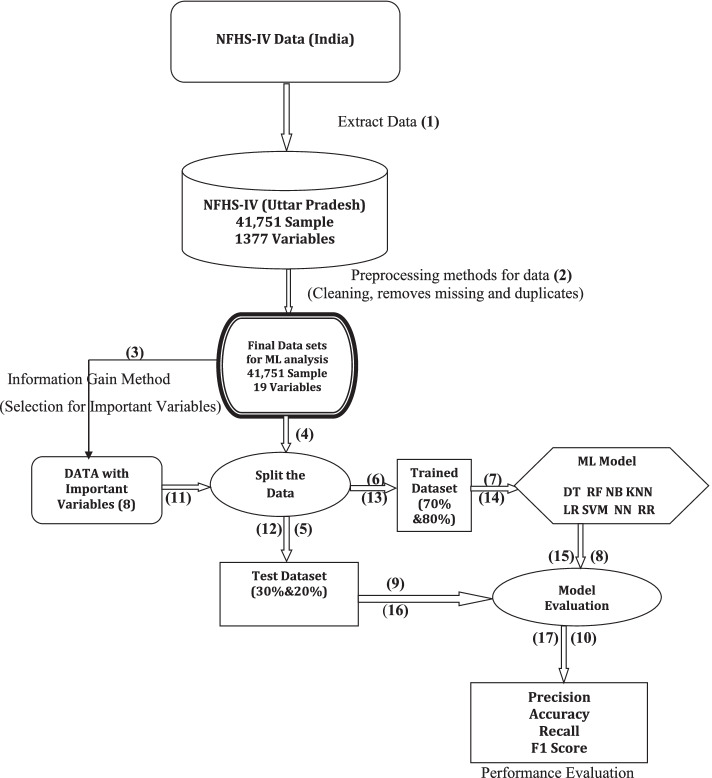
Overview of the proposed framework of machine learning for under-five child mortality data

This study’s methods have been explained step by step through a framework for under-five mortality prediction. The data analysis of this study was performed in various steps. Firstly, the multivariate logistic regression analysis was performed to find the important factors (*p* < 0.05) thereafter machine learning model’s approaches were applied to the dataset. The explanations of the machine learning frameworks are portrayed in Fig. 2. All the analyses of the data were conducted using Python 3.3, STATA 16.0, and SPSS-27 software.

### Importance of ML methods over traditional methods

A study has shown that a machine learning framework can be used to detect significant risk factors of under-five mortality and that deep learning techniques are superior to logistic regression for the classification of child survival [10]. Machine learning models can accurately predict neonatal, perinatal, and infant mortality [11–13]. Several studies done to predict the bankruptcy of banks have shown that intelligent techniques (specifically ANN) seem to work more effectively than statistical techniques. ANN and KNN methods perform more effectively than traditional methods [14].

### Dataset

National Family Health Survey (NFHS-IV) is a large-scale, multi-round cross-sectional, national representative survey conducted in households throughout the Indian states and union territories and is one of the most extensive data collection methods to help keep records across India. The reports are summarized from district-wise to state-wise. The survey collects extensive information on population, health, and nutrition, with an emphasis on women and young children. In this study, we have used secondary data from the NFHS-IV survey of Uttar Pradesh. We have used the target group data of under-five children of Uttar Pradesh. This dataset has records for every woman interviewed whose child was born in the past five years preceding the survey. It contains information related to the mother’s pregnancy, postnatal care, and health. This file was used to obtain information related to child health indicators such as immunization coverage, vitamin A supplementation, recent occurrences of diarrhoea, fever, and cough for young children, and treatment of childhood diseases. A total of 1377 variables were available in this dataset. There was a total of 41,751 samples/individuals, out of which under-five mortality was 2830.

### Study variables

According to an analytical framework for child survival in developing countries [15], we have used 19 (out of 1377 variables) most important variables that were related to under-five mortality, as most of the variables were not useful for this study. Due to missing values, only 15 variables were used for the analysis, which included the outcome/target variable. A missing value is defined as a variable that should have a response but does not have a response either because the question was not asked (due to interviewer error) or the respondent did not want to answer. The outcome/target (dependent) variable was under-five mortality which is known as the death of a child before completing 59 months.

The predictor (independent) variables considered in this study were mothers’ educational level, births in the last five years, any exposure, currently breastfeeding, total number of living children, wealth index, mass media exposure (MXP), survival time, the total number of children ever born, desire for more children, sex of the child, child-size at birth, ANC visits and birth order.

### Data pre-processing

After making the final dataset, the next step was to pre-process the data by using various methods. In this step, the duplicates and missing variables were removed using the predictive mean matching method. Thereafter, all string and categorical variables were transformed into numerical values.

An important point in data pre-processing is the need to balance the target or outcome variable. In the dataset, the numbers of under-five mortality were highly skewed as compared to live children (38,921 live children vs 2830 under-five mortality). A random over-sampling method was used to balance the target (dependent), after which a ratio of 50:50 was obtained as compared to the early ratio of 93:7.

### Feature selection

The idea of feature selection is about ranking the major risk factors from the dataset according to their importance. This is based on the calculation of the information gain values for each of the selected variables. In this study, we have used a random forest model to find the risk factors or important features that have a major contribution to child mortality. The higher information gain values tell us important variables that are highly correlated with the class of variable. We randomly selected the top eight ranked information values, which we used in the model building later.

### Model building

#### Data Splitting

In this step, we split the datasets into trained and test data. 70% of the trained data are used for the model classification and 30% of the data for model evaluation. Again, we will split the datasets into trained and tested (80% and 20% respectively) for a clear idea of a classification model. All the independent features needed to be changed in one-hot encoding to build better predictive models. In this study, the dependent variable was binary, i.e., dead/alive. We then used various suitable machine learning models, namely decision tree, random forest, Naïve Bayes, KNN model, logistic regression, SVM, neural network, and ridge classifier.

### Decision Tree (DT)

The decision tree is one of the most intuitive and straightforward techniques in machine learning based on the divide and conquers paradigm [16]. In a decision tree technique, tests (on input patterns) and categories (of patterns) are used as inner and leaf nodes, respectively. This technique also assigns a class number to an input array by filtering the array down via the tests in the tree [12].

### Random Forest (RF)

The random forest algorithm takes hyper-parameters, identifying the number of trees and the maximum depth of each tree. The random forest is a combination of learning approaches for the classification in machine learning and uses a vast collection of de-correlated decision trees [17].

### Support Vector Machine (SVM)

The SVM is a supervised machine learning technique for analyzing and recognizing patterns of data [18]. New observations are predicted based on class and the side of the partition they fall in. The SVM is the nearest data point to the hyperplane that divides the classes.

### Logistic Regression (LR)

Logistic regression is a statistical classification probabilistic model that predicts the probability of occurrence of an event. The logistic regression model is used to model the categorical dependent variable and a dichotomous categorical outcome or feature. It is a binary (multiple) model used to predict binary (multiple) responses [16]. The predictors need to be independent and significantly associated with the outcome variables [19].

### Naive Bayes (NB)

Naive Bayes is a simple machine learning algorithm based on the Bayes theorem, and it has a necessary assumption that the attributes are conditionally independent for the given class. Naive Bayes gives competitive classification accuracy [20]. Naïve Bayes is widely applied because of its computational efficiency and desirable features [21].

### K- Nearest Neighbours (KNN)

The KNN is a simple and effective non-parametric method of classification, and it is effective in many cases [22]. To classify the data record ‘t’, its ‘k’ nearest neighbour is collected, forming a neighbourhood ‘t’. Most points among the data records in the neighbourhood is mainly used to decide the classification for ‘t’ with or without consideration of distance-based weighting. While applying the KNN, we choose an appropriate value for ‘k’, and the classification success depends on this value. There are several methods of determining k values, but the simplest one is to run the algorithm many times with varying k values and choose the best performance [23].

### Neural network

Neural networks reflect the human brain's behavior and allow computer programs to find patterns and solve common problems in machine learning, artificial learning, and deep learning. ANN comprises a node layer that contains an output layer, an input layer, and one or more hidden layers [24]. Each node connects to another and has an associated weight and threshold. If the output of an individual node exceeds the given threshold value, that node is activated and sends data to the next layer of the network.

### Ridge regression

Ridge regression is a method for estimating the multiple-regression models' coefficients when the independent variables are highly correlated. This method was developed as a possible solution to the imprecision of least squares estimators with multi-collinearity among the independent variables in the linear regression model [25]. Ridge parameter estimates are more precise because their mean square error and variance are smaller than the least square estimators.

### Evaluation for predictive models

In this study, to predict the best model for under-five mortality, evaluation was conducted by various indices such as confusion matrix, sensitivity, specificity, precision, accuracy, F1 score, negative predictive value, Cohen’s Kappa values, and AUROC. All the details as given below:

### Confusion matrix

The confusion matrix visualizes the actual and predicted class accuracies [26]. To examine the performance of the classification algorithm, the confusion matrix compares the predicted classification versus actual classification through the measures; true positive (TP), false positive (FP), true negative (TN), and false-negative (FN), and the formulas are given below.

- **True positive (TP)** – The model correctly predicts positive class in the outcome.
- **True negative (TN)** –The model correctly predicts negative class in the outcome.
- **False-positive (FP)** – The model incorrectly predicts a positive class in the outcome.
- **False-negative (FN)** –The model incorrectly predicts a negative class in the outcome.
- **Sensitivity –** Sensitivity is the test to measure correctly positive predicted events out of a total number of positive events. This gives us the value of how many positives are predicted out of total positive classes. This is known as recall and can be calculated by the given formula:
  $$\mathbf{S}\mathbf{e}\mathbf{n}\mathbf{s}\mathbf{i}\mathbf{t}\mathbf{i}\mathbf{v}\mathbf{i}\mathbf{t}\mathbf{y}/\mathbf{R}\mathbf{e}\mathbf{c}\mathbf{a}\mathbf{l}\mathbf{l}=\frac{\mathbf{T}\mathbf{P}}{\mathbf{T}\mathbf{P}+\mathbf{F}\mathbf{N}}$$

**Specificity –** Specificity is the measure that tells us the proportion of correctly predicted negative outcomes among all total negative outcomes. It can be calculated by the given formula:

$$\mathbf{S}\mathbf{p}\mathbf{e}\mathbf{c}\mathbf{i}\mathbf{f}\mathbf{i}\mathbf{c}\mathbf{i}\mathbf{t}\mathbf{y}=\frac{\mathbf{T}\mathbf{N}}{\mathbf{T}\mathbf{N}+\mathbf{F}\mathbf{P}}$$

**Precision –** Precision is the correct events divided by the total number of positive events that the classifier predicts. This is also known as positive predictive value. In this study, it was used to check the model output from the given formula below and it was calculated from the confusion matrix:

$$\mathbf{P}\mathbf{r}\mathbf{e}\mathbf{c}\mathbf{i}\mathbf{s}\mathbf{i}\mathbf{o}\mathbf{n}/\mathbf{P}\mathbf{P}\mathbf{V}=\frac{\mathbf{T}\mathbf{P}}{\mathbf{T}\mathbf{P}+\mathbf{F}\mathbf{P}}$$

**Negative predictive value –** The negative predictive value is defined as the number of true negatives divided by the total number of people who test negative.

$$\mathbf{Negative}\boldsymbol\;\mathbf{predictive}\boldsymbol\;\mathbf{value}\boldsymbol\;\boldsymbol=\frac{\mathbf{TN}}{\mathbf{TN}\boldsymbol\;\boldsymbol+\boldsymbol\;\mathbf{FN}}$$

**Accuracy –** Accuracy is the percentage of true events among the total number of cases tested. In this study, it was used to determine model efficacy and measure from the confusion matrix.

$$\mathbf{A}\mathbf{c}\mathbf{c}\mathbf{u}\mathbf{r}\mathbf{a}\mathbf{c}\mathbf{y}=\frac{\mathbf{T}\mathbf{P}+\mathbf{T}\mathbf{N}}{\mathbf{T}\mathbf{P}+\mathbf{T}\mathbf{N}+\mathbf{F}\mathbf{P}+\mathbf{F}\mathbf{N}}$$

**F1 score—**The inverse relationship between accuracy and recall is the F1 score or the F test. The higher value of the F1 score predicts a better model. The harmonic mean of recall and accuracy is determined as.

$$\mathbf F1\;\mathbf s\mathbf c\mathbf o\mathbf r\mathbf e=\frac{2\mathbf T\mathbf P}{2\mathbf T\mathbf P+\mathbf F\mathbf N+\mathbf F\mathbf P}$$

**Cohen’s Kappa—**Cohen’s Kappa is a coefficient used to assess the performance of the binary classification model [27]. It is a very useful evaluation statistic coefficient when working with imbalanced data. Cohen’s Kappa (*k*) is calculated by the given formula:

$${\varvec{k}}\boldsymbol{ }=\frac{{{\varvec{p}}}_{{\varvec{o}}}-{{\varvec{p}}}_{{\varvec{e}}}}{1-{{\varvec{p}}}_{{\varvec{e}}}}$$

where ${p}_{o}$ is the overall accuracy of the model and is the measure of the agreement between the model predictions and the actual class values as if happening by chance? It can range from 0 to 1, with 0 representing no agreement and 1 representing the perfect agreement between classes.

### Area under Receiver Operator Characteristic (AUROC) Curve

The Receiver Operator Characteristic curve is the probability curve that shows the relationship between sensitivity and specificity. This curve is the most used metric for binary classification outcomes. The Field under the ROC shows how well the probabilities are segregated from the negative classes by the positive classes. When the AUC value is close to 1, the model prediction indicates better, while the value near 0 indicates bad model efficiency. In this study, we use this measure for the model’s efficiency.

### Precision-recall curve

The precision-recall curve is a combination of sensitivity (x-axis) and precision(y-axis). It’s used as an alternative to roc curves [28]. The high precision relates to a low false positive rate, while high recall is related to low false. The maximum area under the curve denotes both high precision and high recall. The highest score for both measures indicates that the classifier is producing results that are mostly positive (high recall) and accurate (high precision).

## Results

The multivariate logistic regression analysis was applied to predict the important factors in under-five mortality data. Table 1 shows births in the last five years, breastfeeding status, sex of the child, number of living children, child size at birth, sex of the child, birth order, survival time, children ever born, and desire for more children were important factors for under-five mortality.

**Table 1:** Multivariate Logistic Regression Analysis for predicting the factors for under-five mortality data

**Background Characteristics**		**Standard Error**	**P -value**	**Odds Ratio**	**Confidence Interval**	
**Births in the last five years**					**Lower**	**Upper**
No births (Ref)						
One birth		0.03	0.00	0.39	0.33	0.44
= > 2 birth		0.01	0.00	0.17	0.14	0.20
**Breastfeeding status**						
No (Ref)						
Yes		0.21	0.00	3.71	3.33	4.14
**Exposure**						
Fecund (Ref)						
Pregnant		0.08	0.66	1.03	0.89	1.20
Postpartum amenorrheic		0.06	0.06	0.89	0.78	1.01
Infecund, menopausal		0.15	0.68	0.94	0.68	1.28
**Sex of child**						
Female (Ref)						
Male		0.06	0.01	1.14	1.03	1.26
**Living children**						
No Children (Ref)						
One Child		0.0012	0.00	0.01	0.01	0.01
> 2 Children				1.00		
**Child Size**						
Large (Ref)						
Average		0.12	0.00	1.60	1.38	1.85
Smaller		0.07	0.05	0.85	0.72	1.00
**ANC**						
No (Ref)						
Yes		0.23	0.00	2.09	1.69	2.58
**Birth order**						
One birth order (Ref)						
Two birth order		0.18	0.00	2.05	1.73	2.44
> 2 Birth order		0.11	0.50	1.07	0.88	1.31
**Education level**						
No education (Ref)						
Primary		0.07	0.78	0.98	0.85	1.13
Secondary		0.06	0.79	0.98	0.87	1.12
Higher		0.12	0.60	0.93	0.72	1.21
**Wealth Index**						
Poor (Ref)						
Middle		0.07	0.44	0.95	0.83	1.08
Rich		0.09	0.11	1.13	0.97	1.31
**MXP**						
No exposure (Ref)						
Any exposure		0.06	0.57	0.97	0.86	1.08
**Time in months**						
0–11 (Ref)						
12–23		0.06	0.00	0.70	0.59	0.84
24–35		0.06	0.00	0.72	0.60	0.86
36–47		0.06	0.00	0.65	0.54	0.77
48–59		0.07	0.01	0.78	0.65	0.93
**Children ever born**						
One Child (Ref)						
Two Children		1.48	0.00	11.47	8.91	14.76
> Two Children				1.00		
**Desire for more children**						
One Child (Ref)						
Two Children		0.05	0.00	0.57	0.48	0.67
> Two Children		0.04	0.00	0.64	0.56	0.73
**Constant**						
		2.07	0.00	11.62	8.20	16.47

The machine learning models, namely decision tree, random forest, Naïve Bayes, KNN, logistic regression, SVM, neural network, and ridge classifier were applied to build a predictive model of under-five mortality. A comparison of 70% training and 30% validation, 80% training, and 20% validation was done by eight machine learning models including various evaluation measures with and without important data factors.

**Fig. 3:**
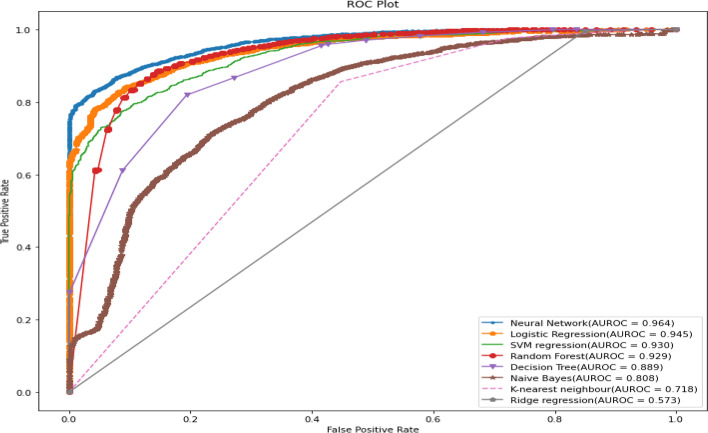
ROC curve for machine learning models in predicting under-five mortality with all factors (70/30 Ratio)

**Fig. 4:**
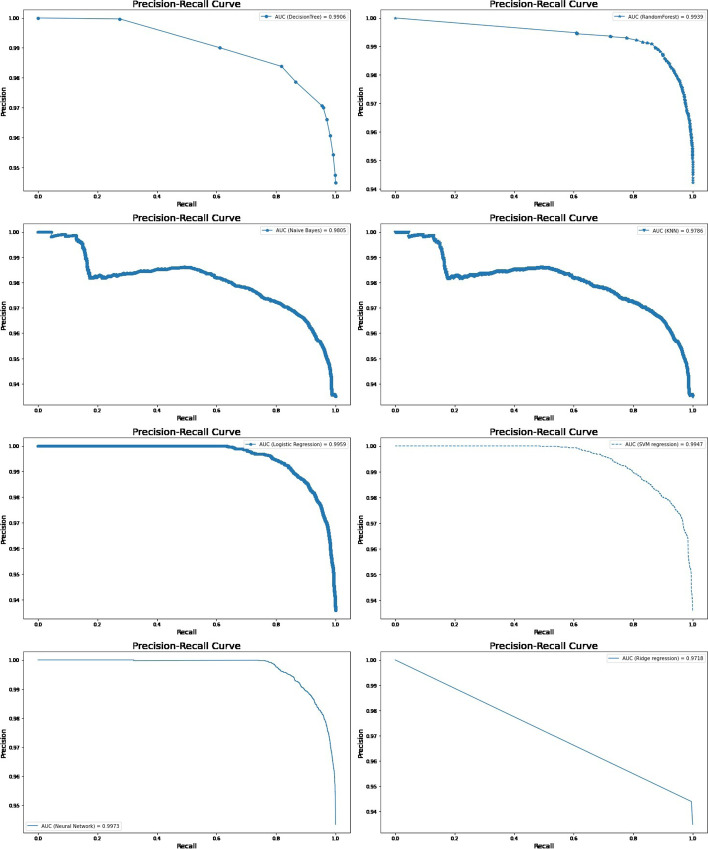
Precision-Recall curves for machine learning models in predicting under-five mortality with all factors (70/30 Ratio)\

All predictive models of under-five mortality were applied to training data of 70% with all factors. The models were tested on test data 30%. The performance of predictive models was evaluated and compared using various metrics namely confusion matrix, sensitivity, specificity, precision, accuracy, F1 score, negative predictive value, Cohen’s Kappa values, and AUROC curve. The result of the model evaluation is shown in Table 2 for 70% of the trained data. The results showed that the neural network model had predicted under-five mortality at 95.96% highest accuracy with a recall (81.03%), precision (51.83%), F1 score (62.68%), and Cohen’s Kappa value (0.60). The result indicates that the neural network model was the best predictive model for under-five mortality compared to other predictive models. The ROC curve is shown in Fig. 3, and the precision-recall curve is shown in Fig. 4. Both curves of the neural network model show the highest AUROC (96.4%) and highest precision-recall curve (99.7%), again indicating that it is the best predictive model among all models. The second-best model shows logistic regression analysis with 94.5% AUROC and 99.6% precision-recall curve value.

**Table 2:** The performance of the prediction models with all factors based on various indices for two ratios

Train/test ratios	Measures	Decision tree	Random forest	Naive Bayes	K-Nearest neighbour	Logistic regression	SVM regression	Neural network	Ridge regression
70/30	Sensitivity	93.30	72.66	40.00	58.62	64.11	63.27	80.62	71.51
	Specificity	94.74	96.49	94.32	94.10	95.67	95.70	96.47	94.39
	Precision	20.47	48.53	14.95	10.42	35.91	36.52	47.92	15.07
	Accuracy	94.72	95.46	92.99	93.69	94.52	94.48	95.86	94.08
	F1 Score	33.57	58.19	21.77	17.69	46.03	46.31	60.11	24.90
	Negative Predictive value	99.90	98.73	98.44	99.49	98.60	98.52	99.20	99.58
	Cohen’s Kappa values	0.32	0.56	0.19	0.17	0.44	0.44	0.58	0.24
80/20	Sensitivity	92.91	75.41	41.55	60.78	65.00	65.55	79.27	71.31
	Specificity	94.80	96.62	94.35	94.13	95.79	95.87	96.71	94.42
	Precision	21.61	50.55	15.75	11.36	38.10	39.38	51.83	15.93
	Accuracy	94.77	95.69	93.04	93.73	94.61	94.68	95.96	94.08
	F1 Score	35.07	60.53	22.84	19.14	48.04	49.20	62.68	26.05
	Negative Predictive Value	99.88	98.85	98.45	99.49	98.57	98.55	99.05	99.55
	Cohen’s Kappa values	0.33	0.58	0.20	0.17	0.45	0.47	0.60	0.25

**Fig. 5:**
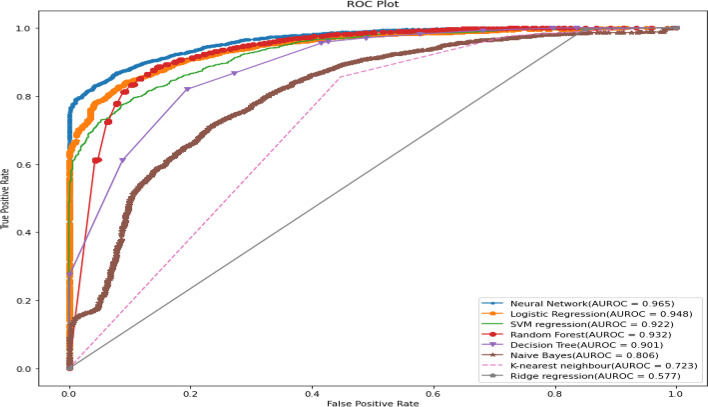
ROC curve for machine learning models in predicting under-five mortality with all factors (80/20 Ratio)

**Fig. 6:**
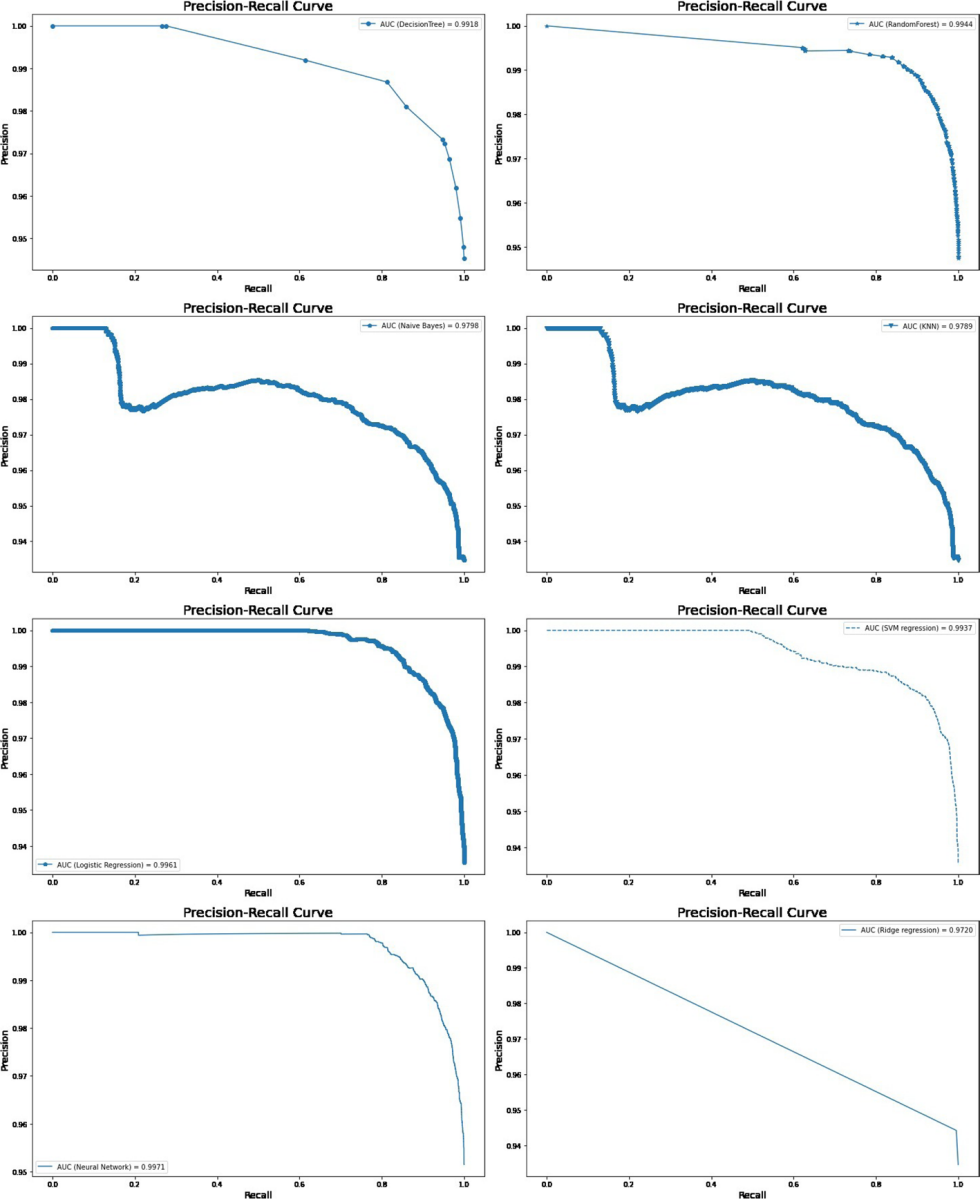
Precision-Recall curve for machine learning models in predicting under-five mortality with all factors (80/20 Ratio)

Again, all predictive models of under-five mortality were applied to training data of 80% with all factors to get a better idea regarding the accuracy or efficacy of the model. The result of the model evaluation is shown in Table 2 for 80% of trained data. The result indicated that the neural network model was the best predictive model for under-five mortality compared to other predictive models. The result findings found that the neural network model has predicted under-five mortality at 95.96% highest accuracy with recall (79.27%), precision (51.83%), F1 score (62.68%), and Cohen’s Kappa value (0.60). The ROC curve is shown in Fig. 5, and the precision-recall curve is shown in Fig. 6. The curve of the neural network model shows the highest AUC (93.87%), and highest precision-recall curve (99.7%), indicating it is the best predictive model among the models. The second-best model shows the logistic regression model with 94.8% AUROC and 99.6% precision-recall curve value.

After that, we used a random forest model to find the risk factors or important features that had a major contribution to the mortality of under-five children. We used the information gain rank method of random forest to check feature importance concerning its predictive power.

**Fig. 7:**
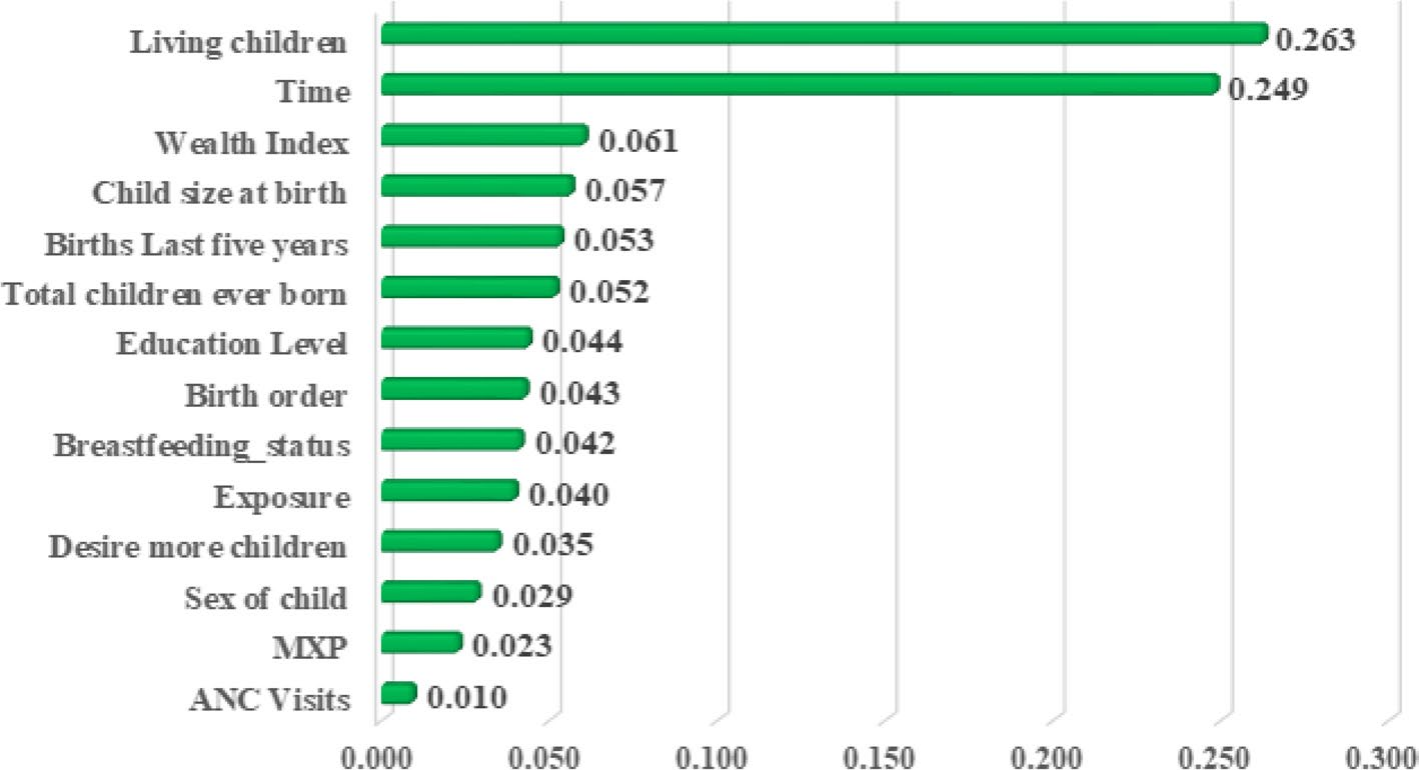
Information gain rank values of the variables under study

We selected only the top eight best features for the model’s accuracy. The result of feature importance is shown in Fig. 7. The result showed that the most important determinants of under-five mortality were the number of living children, survival time, wealth index, child size at birth, birth in the last five years, total children ever born, mother’s education level, and birth order because they were high rank in order. After that, we repeated all procedures with important factors to know the importance of information gain measures or very important features.

All machine learning models, namely decision tree, random forest, Naive Bayes, KNN, logistic regression, SVM, neural network, and ridge classifier were applied to build a predictive model of under-five mortality in training data of 70% with eight important factors.

**Fig. 8:**
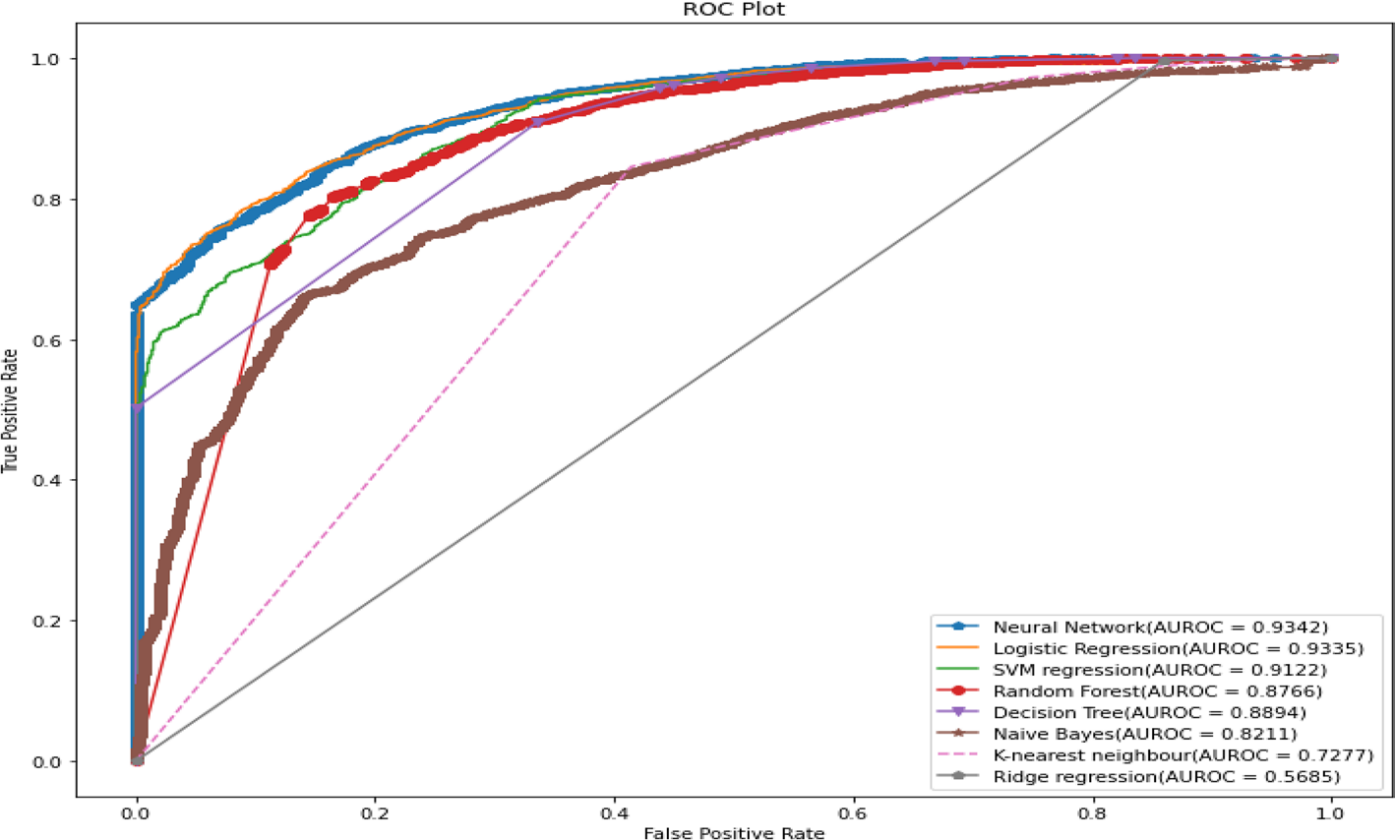
ROC curve for machine learning models in predicting under-five mortality with important factors (70/30) Ratio

**Fig. 9:**
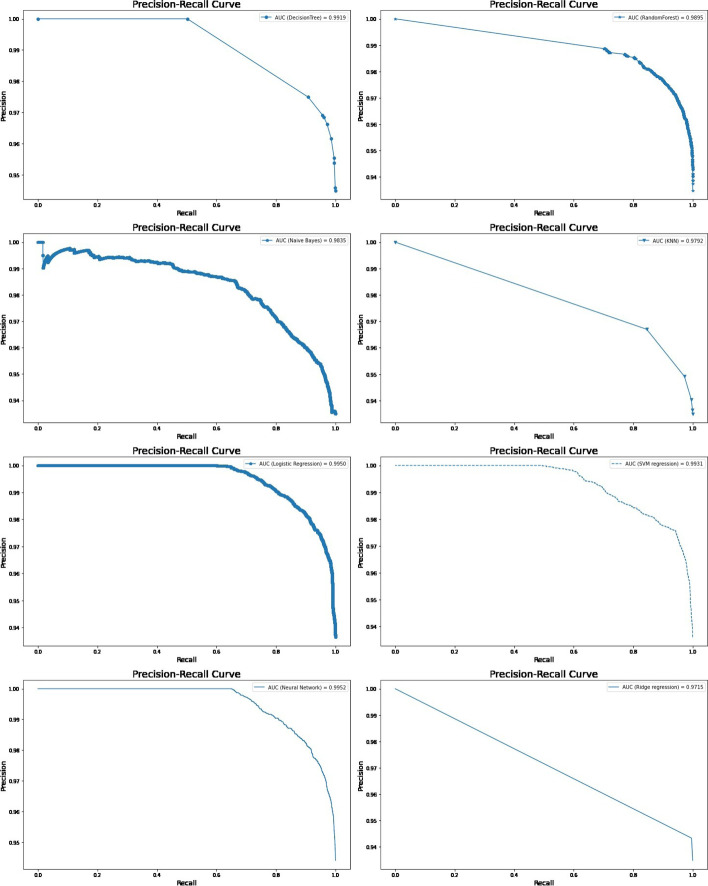
Precision-Recall curve for machine learning models in predicting under-five mortality with important factors (70/30 Ratio)

The models were tested on test data 30%. The result of the model evaluation is shown in Table 3 for 70% of trained data. The result indicates that the neural network model was the best predictive model for under-five mortality compared to other predictive models. The result showed that the neural network model had predicted under-five mortality at 95.31% highest accuracy with recall (81.03%), precision (36.64%), F1 score (50.46%), and Cohen’s Kappa value (0.48). The ROC curve is shown in Fig. 8, and the precision-recall curve is shown in Fig. 9. The curve of the neural network model showed the highest AUC (93.51%), and the precision-recall curve (99.5%) indicated it is the best predictive model among the models. The logistics regression model indicated the best second model with 93.3% AUROC and 99.5% precision-recall curve value.

**Table 3:** The performance of the prediction models with important factors based on various indices for two ratios

Train/test ratios	Measures	Decision tree	Random forest	Naive Bayes	K-Nearest neighbour	Logistic regression	SVM regression	Neural network	Ridge regression
70/30	Sensitivity	83.90	58.40	21.83	57.78	70.59	70.23	81.03	71.43
	Specificity	95.53	96.05	93.73	94.04	95.55	95.55	95.75	94.33
	Precision	33.21	42.16	5.27	9.56	33.82	33.82	36.64	14.09
	Accuracy	95.23	94.28	92.60	93.65	94.77	94.75	95.31	94.04
	F1 Score	47.59	48.97	8.49	16.40	45.73	45.66	50.46	23.54
	Negative Predictive value	99.56	97.91	98.68	99.51	99.02	99.00	99.40	99.61
	Cohen’s kappa values	0.46	0.46	0.06	0.15	0.44	0.44	0.48	0.22
80/20	Sensitivity	84.3	58.40	21.21	60.00	71.79	71.64	71.51	71.68
	Specificity	95.60	96.05	93.70	94.01	95.67	95.62	96.25	94.36
	Precision	34.43	42.16	5.13	9.34	35.90	35.16	45.05	14.84
	Accuracy	95.23	94.28	92.55	93.67	94.89	94.85	95.29	94.05
	F1 Score	48.89	48.97	8.26	16.16	47.86	47.17	55.28	24.58
	Negative Predictive Value	99.55	97.91	98.67	99.56	99.01	99.03	98.74	99.59
	Cohen’s kappa values	0.47	0.46	0.06	0.15	0.46	0.45	0.53	0.23

**Fig. 10:**
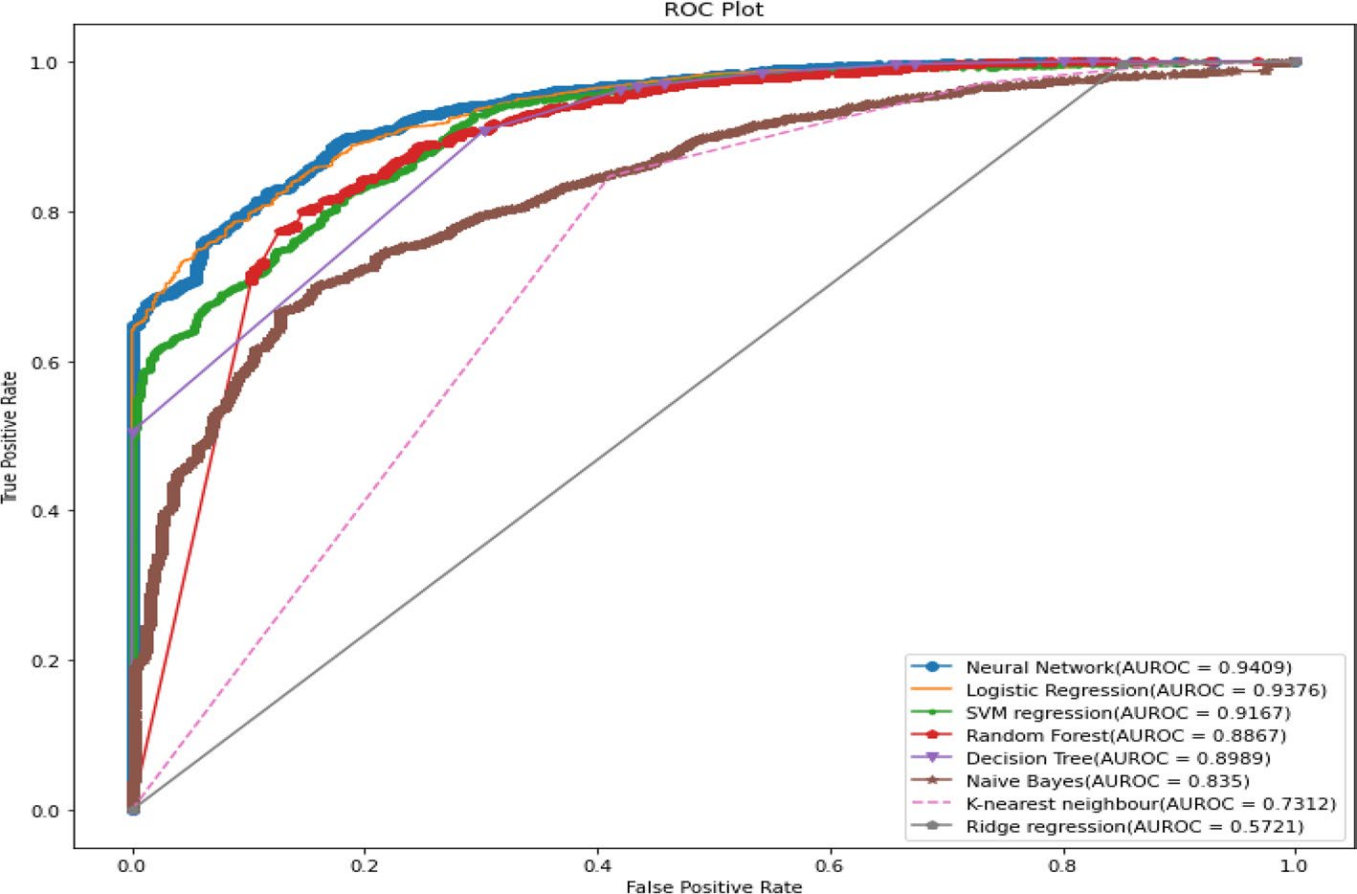
ROC curve for machine learning models in predicting under-five mortality with important factors (80/20 Ratio)

**Fig. 11:**
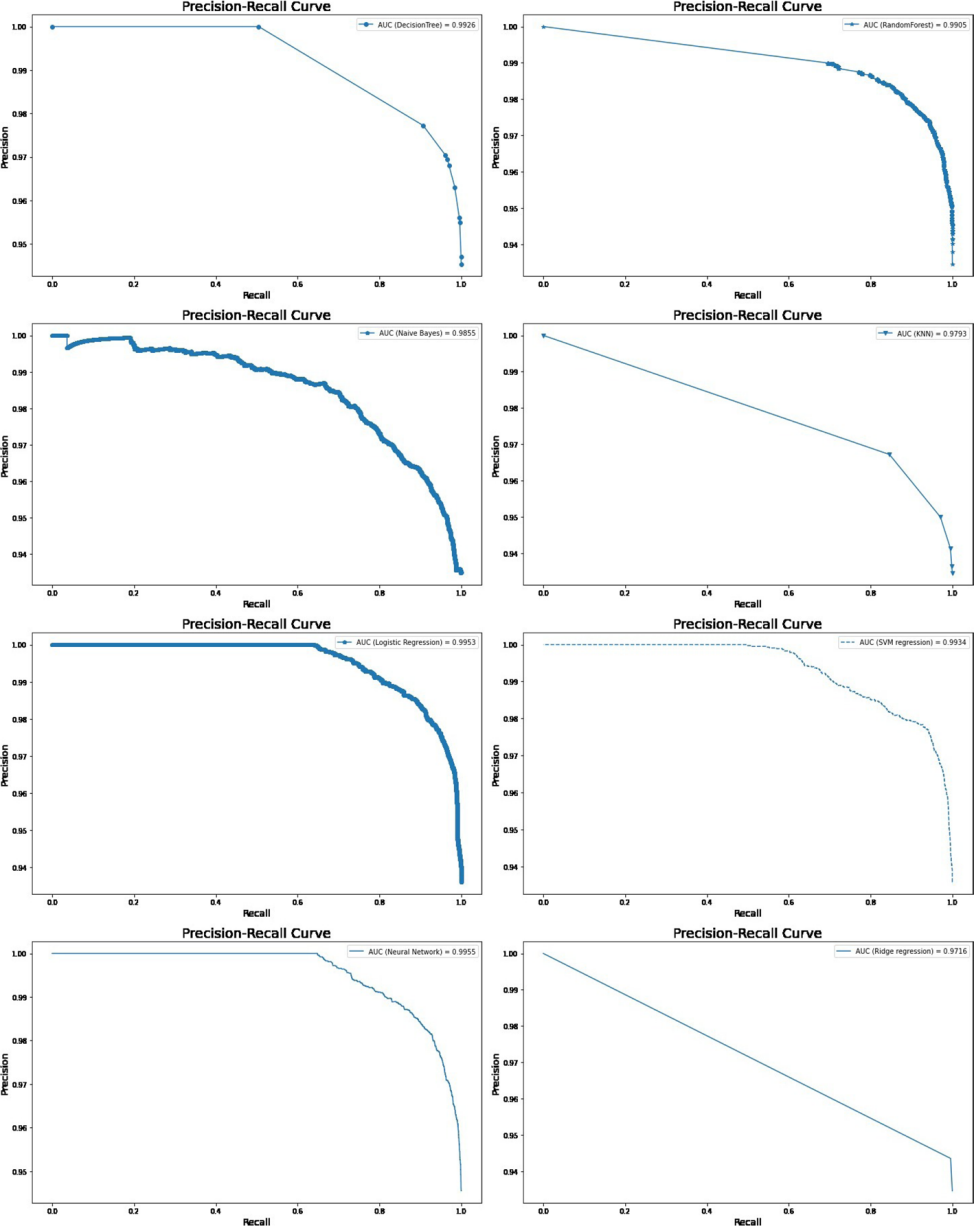
Precision-Recall curve for machine learning models in predicting under-five mortality with important factors (80/20 Ratio)

Again, all predictive models of under-five mortality were applied to training data of 80% with eight important factors. The models were tested on test data 20%. The result of the model evaluation is shown in Table 3 for 80% of trained data. The result found that the neural network model predicted under-five mortality at 95.29% highest accuracy with recall (71.51%), precision (45.05%), F1 score (55.28%), and Cohen’s Kappa value (0.53), indicating it is the best predictive model among the models. The ROC curve is shown in Fig. 10, and the precision-recall curve is shown in Fig. 11. The curve of the neural network model shows the highest AUC (93.95%) and the precision-recall curve (99.5%) is the best predictive model among the models. The second-best model was a logistic regression with 94.8% AUROC and 99.5% precision-recall curve value. Finally, the result declared that the neural network classifier model is the most accurate model for predicting under-five mortality in the predictive analytics structure. The result also confirms that the machine learning model shows better output accuracy than the traditional statistical model and the information gain ranked method predicts the under-five mortality factors.

## Discussion

This study predicts the important factors of under-five mortality using logistic regression analysis and a machine learning model. This study evaluated the importance of machine learning techniques in predicting the factors of under-five mortality. This is the first study that used machine learning techniques in high under-five mortality data of an Indian state Uttar Pradesh, to predict under-five mortality. To find better accuracy of machine learning models, we applied two different ratios i.e. 70/30 and 80/20 and we observed that the 70/30 ratio was the appropriate ratio for the model and this result is justified by previous studies [29, 30]. This study showed that the neural network predictive model is better than another predictive model for predicting the factors of under-five mortality data. Concerning the predictive analysis, the prediction accuracy was (95.29% to 95.96%), recall (71.51% to 81.03%), precision (36.64% to 51.83%), F1 score (50.46% to 62.68%), Cohen’s Kappa value (0.48 to 0.60) AUROC (93.4% to 96.5%) and precision-recall curve (99.5% to 99.7%) in the neural network model compared to other predictive models. The study also shows that logistic regression analysis is close to the neural network method in this data and the model seems to perform with near similar accuracy. However, we were unable to demonstrate that one technique is better than the other. The various research articles found that neural networks were superior to logistic regression [31–33]. The articles found no differences between LR, and neural networks and some articles found that logistic regression was better than neural networks [34, 35]. It may not be possible to determine which model is superior to the other in each dataset but the neural network’s ability to detect the complex nonlinear relationship and all possible interactions between predictor variables. The neural network gives impressive results from an overfitted model including various free parameters while logistic regression has less potential for overfitting. All variables in a dataset are rarely useful for developing machine learning models. Adding maximum variables in the analysis reduces the competence and accuracy of the models. Thus, feature selection is an important tool in machine learning to find the important factors that are useful in machine learning models.

The feature information gain method showed that the number of living children, time, wealth index, child size at birth, birth in the last five years, total children ever born, mother’s education level, and birth order are the top eight important predictors for under-five mortality.

Various studies also confirmed that these factors are crucial for under-five mortality [3–39]. From this study, we can confirm that the wealth index was one of the important factors for under-five mortality, which is in line with other studies [40–42]. This study found time was a significant factor in under-five mortality [43, 44]. This study’s findings observed that the mother's education level was one of the major under-five mortality factors. Previous studies also confirmed that the mother’s education played an important role in reducing the risk of under-five mortality [45–48]. This may be because educated mothers might have better knowledge about the health services, care, and immunization of their children.This study found that the number of births in the last five years and birth order is an important factor related to under-five mortality.

Previous studies have shown that the likelihood of under-five mortality increases with the number of births in the last five years and the total number of children ever born [49–51]. These results are like the study reported using the ML approach [52] and traditional methods [53].

It has been shown that child size at birth plays a significant role in under-five mortality and a similar result was found in previous research also [54, 55]. A study reported that neural network has higher predictive accuracy for under-five mortality prediction [56]. The neural network model is stable in forecasting infant mortality rates as compared to the conventional logistic regression model and performs more accurately in predicting five-year mortality also [57, 58].

This approach can predict and simulate the mortality rates in the human population and make accurate predictions of mortality risk for most preterm infants [59, 60]. Previous research also confirms that machine learning model methods are better than traditional analysis methods [61, 62]. A previous study predicted that machine learning models are more suitable for finding the factors of infant mortality and confirming better goodness of fit in most critical groups [63]. Moreover, machine learning models are very valuable in predicting health studies that lead to healthier and more suitable policy decisions.

### Study limitation

This study cannot be complete without its limitation because we have used machine learning models, unlike statistical models. The machine learning model's result comes without any coefficient and odds ratio compared to the statistical model and is difficult to understand how much and in which direction, factors affect the outcome. Another limitation is that we need to decide our research hypothesis in the study, but machine learning models cannot frame research hypotheses for prediction and classification both. The results of the study are based on NFHS-IV questionnaires’ data. It is not a specific study, nor has precise objectives related to under-five mortality. There were various missing variables in the dataset and those variables were not included in the study.

## Conclusion

The objective of this study was to apply the various Machine Learning models to under-five mortality data.

This study explains the ML accuracy and predicts the important factors related to under-five mortality.

The neural network model performed best in predicting under-five mortality with the highest accuracy compared to this study's other machine learning models. The study also indicates that logistic regression analysis can be useful in predicting the mortality of under-five morality with some limitations. However, this study also highlighted that some of the variables have an equally significant impact on under-five mortality in both LR and ML models. The number of children, survival time, child size at birth, birth in the last five years, the total number of children ever born, and birth order were found to be the most important factors for under-five mortality. The machine learning models provide some important factors that may add to analysis capabilities compared to other traditional statistical models. These models may be helpful for the analysis of high-dimensional data for health research.

## Data Availability

The data of the National Family Health Survey is available online. The International Institute for Population Sciences (IIPS), Mumbai website is the nodal agency for the NFHS-4 survey. This data is freely available to access for research anyone after registration. (http://rchiips.org/nfhs/nfhs4.shtml). The source code adds as supplementary file in this article.
